# Jump performance and movement quality in 7- to 15-year-old competitive alpine skiers: a cross-sectional study

**DOI:** 10.1080/07853890.2024.2361254

**Published:** 2024-06-04

**Authors:** Jonas Hanimann, Nadine Raschle, Nathan E. Schmid, Björn Bruhin, Walter O. Frey, Johannes Scherr, Eling D. de Bruin, Jörg Spörri

**Affiliations:** a Sports Medical Research Group, Department of Orthopaedics, Balgrist University Hospital, University of Zurich, Zurich, Switzerland; b University Centre for Prevention and Sports Medicine, Department of Orthopaedics, Balgrist University Hospital, University of Zurich, Zurich, Switzerland; c Department of Health Sciences and Technology, ETH Zürich, Zurich, Switzerland; d Swiss-Ski, Worblaufen, Switzerland; e Hirslandenklinik, Zurich, Switzerland; f Department of Health, OST – Eastern Switzerland University of Applied Sciences, Switzerland

**Keywords:** Injury prevention, skiing, athletes, youth sports, exercise test, physical fitness

## Abstract

**Introduction:**

Injury rates in competitive alpine skiing are high. With current methods, identifying people at risk is expensive and thus often not feasible at the youth level. The aims of this study were (1) to describe the jump performance and movement quality of youth competitive alpine skiers according to age and sex, (2) to compare the jump distance among skiers of different sexes and movement quality grades, and (3) to assess the inter-rater grading reliability of the qualitative visual movement quality classification of such jumps and the agreement between live and video-based post-exercise grading.

**Materials and Methods:**

This cross-sectional study is based on an anonymized dataset of 301 7- to 15-year-old competitive alpine skiers. The skiers performed two-legged forward triple jumps, whereby the jump distance was measured, and grades were assigned by experienced raters from the frontal and sagittal perspectives depending on the execution quality of the jumps. Furthermore, jumps were filmed and ultimately rated post-exercise. Differences in jump distance between various groups were assessed by multivariate analyses of variance (MANOVAs). Reliability was determined using Kendall’s coefficient of concordance.

**Results:**

The jump distance was significantly greater in U16 skiers than in U11 skiers of both sexes and in skiers with *good* execution quality than in those with *reduced* or *poor* execution quality. Overall, jump distance in U16 skiers significantly differed between female (5.37 m with 95% CI [5.21, 5.53]) and male skiers (5.90 m with 95%CI [5.69, 6.10]). Slightly better inter-rater grading reliability was observed for video-based post-exercise (*strong* agreement) ratings than for live ratings (*moderate* agreement).

**Conclusion:**

In competitive alpine skiers aged 7 to 15 years, jump performance increases with age, and around puberty, sex differences start to manifest. Our results highlight the importance of evaluating both jump distance and movement quality in youth skiers. To improve test-retest reliability, however, a video-based post-exercise evaluation is recommended.

## KEY MESSAGES

In youth competitive alpine skiers, jump performance and movement quality matter, and both should be trained and tested.

A qualitative assessment of movement quality while jumping by experts is a highly scalable and cost-effective approach; however, to ensure sufficient test-retest reliability, the assessment criteria need to be standardised and an additional video-based post-exercise assessment is recommended.

## Introduction

Injury rates in competitive alpine skiing are high, with youth skiers being particularly vulnerable during the growth spurt [1,2]. In particular, anterior cruciate ligament ruptures are a major health problem [3–7]. Absence from sports [1], an increased risk of re-ruptures and consequential diseases are concomitant effects [8,9]. Thus, it is not surprising that injury mechanisms, risk factors and prevention approaches have been extensively studied.

In the context of ACL injuries, poor leg axis stability (i.e. dynamic knee valgus) and stiff landings are well-known injury risk factors [10–13]. The identification of athletes at risk commonly includes biomechanical/visual assessments during dynamic motion tasks such as drop jumps [12–19]. The gold standard procedure for this purpose is an assessment by means of a three-dimensional (3D) motion capture system. However, this method is time-consuming and associated with high costs; thus, it is often not applicable in practical sports settings, particularly in youth athletes [15,16,20,21].

Several approaches to simplify the assessment have been described in the literature, and promising results have been achieved [15–18,22,23]. For example, as reported by Nilstad et al. in a visual live assessment including three repetitions, physiotherapists were able to accurately classify subjects’ knee control (i.e. dynamic knee valgus) during vertical drop jumps as ‘good’, ‘reduced’ or ‘poor’, with ground truth assessed with a motion capture system [17]. Moreover, two-dimensional video analysis has proven valuable in knee valgus assessment and has shown comparable results to three-dimensional motion capture-based assessments [15,16,18].

In the present study, we propose a simple and upscalable test approach to assess jump performance and movement quality simultaneously during two-legged forward triple jumps. Despite being easy to use, the execution of two-legged forward triple jumps is a complex dynamic motion task that, when executed properly, powerfully, and fluently, places high demands in terms of movement coordination and neuromuscular control on athletes. To ensure an applicable assessment setup in real-world settings, a classification based on live observation and video assessment was considered appropriate [17,18]. Hence, for the movement execution evaluation, an all-encompassing rating system with three possible grades was adopted in addition to measuring jump distance. The qualitative criteria catalogue particularly focused on injury prevention-relevant factors of movement quality (stable leg axes, soft landings and superior dynamic postural control, that is, quick and controlled recovery to a stable position after the last landing) [11,13,24].

Accordingly, the aims of this study were (1) to describe the performance and movement quality of youth competitive alpine skiers in two-legged forward triple jumps according to age and sex, (2) to compare the jump distance of skiers of different sexes and movement quality grades, and (3) to assess the inter-rater grading reliability of the qualitative visual movement quality classification of such jumps and the agreement between live and video-based post-exercise grading.

## Materials and methods

### Participants and study design

This cross-sectional study is based on the reuse of an anonymized dataset of 7- to 15-year-old competitive alpine skiers originally collected as part of a physical fitness competition called the ‘Swiss-Ski Summer Challenge’ in 2022. A total of 327 youth skiers participated in this event. Twenty skiers were excluded because they were not able to fulfill the minimal execution criteria (as described below), and six skiers were excluded due to technical issues and lack of video material for post-exercise evaluation. This led to a total sample size of 301 participants being used for this study. The present study was conducted in accordance with Swiss national laws and the guidelines of the Declaration of Helsinki. The reuse of the anonymized dataset was approved by the Cantonal Ethics Committee KEK Zurich (KEK-ZH-NR: 2021-01044). Participant consent was waived because the present study used anonymized data, and the underlying study protocol was therefore judged by the Cantonal Ethics Committee KEK Zurich as not falling under the scope of the Swiss Human Research Act (HRA). The data are reported in line with the STROBE Statement [25].

### Data collection

#### Two-legged forward triple jump test

Participants were asked to position behind the starting line, taking a hip-width stand with the most ventral points of the feet touching the line. There was no starting sign or prescription prior to the start of the test. During the subsequent second and third jumps, participants were asked to perform only one arm swing per jump, land with both feet simultaneously and have as little ground contact time as possible. A jump was considered invalid and, thus, repeated if there were breaks between the individual jumps (no smooth movement execution) or if more than three ground contacts were made (i.e. small jumps or steps other than the task or touching the floor with a body part other than the feet). Finally, the participants had to stand for at least three seconds in a stable and controlled manner after the last jump. A maximum of four trials were allowed; however, repetition was allowed only in the case of invalid jumps, and the test results were not improved. The jump distance was defined as the distance between the starting line and the participants’ most distal ground contact point (i.e. heels) at the final landing position (1 cm, determined by measuring tape).

#### Baseline characteristics

The participants’ date of birth was provided with the event inscription, and age (years) was calculated. In addition, for skiers aged 11 to 15 years, maturity offsets were estimated using the non-invasive estimation Mirwald method [26]. For this purpose, weight was measured by a scale (1 kg, Seca, Hamburg, Germany), and height and sitting height were determined by size measurement (1 cm, determined by measuring tape).

#### Movement control and execution grading

For each valid jump test, a grade was assigned. The rating criteria were maintaining stable leg axes in the frontal plane throughout all jumps; soft landing with adequate involvement of the ankle, knee, and hip joint from the sagittal plane; and fast attainment of a stable position after landing. Thus, increased dynamic knee valgus, stiff landings with insufficient shock absorption, and excessive compensatory movements or involvement of the arms to regain a stable position led to grade deductions. Grades 1, 2 and 3 were related to *poor*, *reduced* and *good* task execution, respectively, based on the criteria. The rating was performed from both the frontal and sagittal perspectives, as well as live and post-exercise based on recordings from a high-speed video camera at 120 Hz (Vicon Vue, Oxford Metrics).

#### Descriptive data

**Figure 1.**
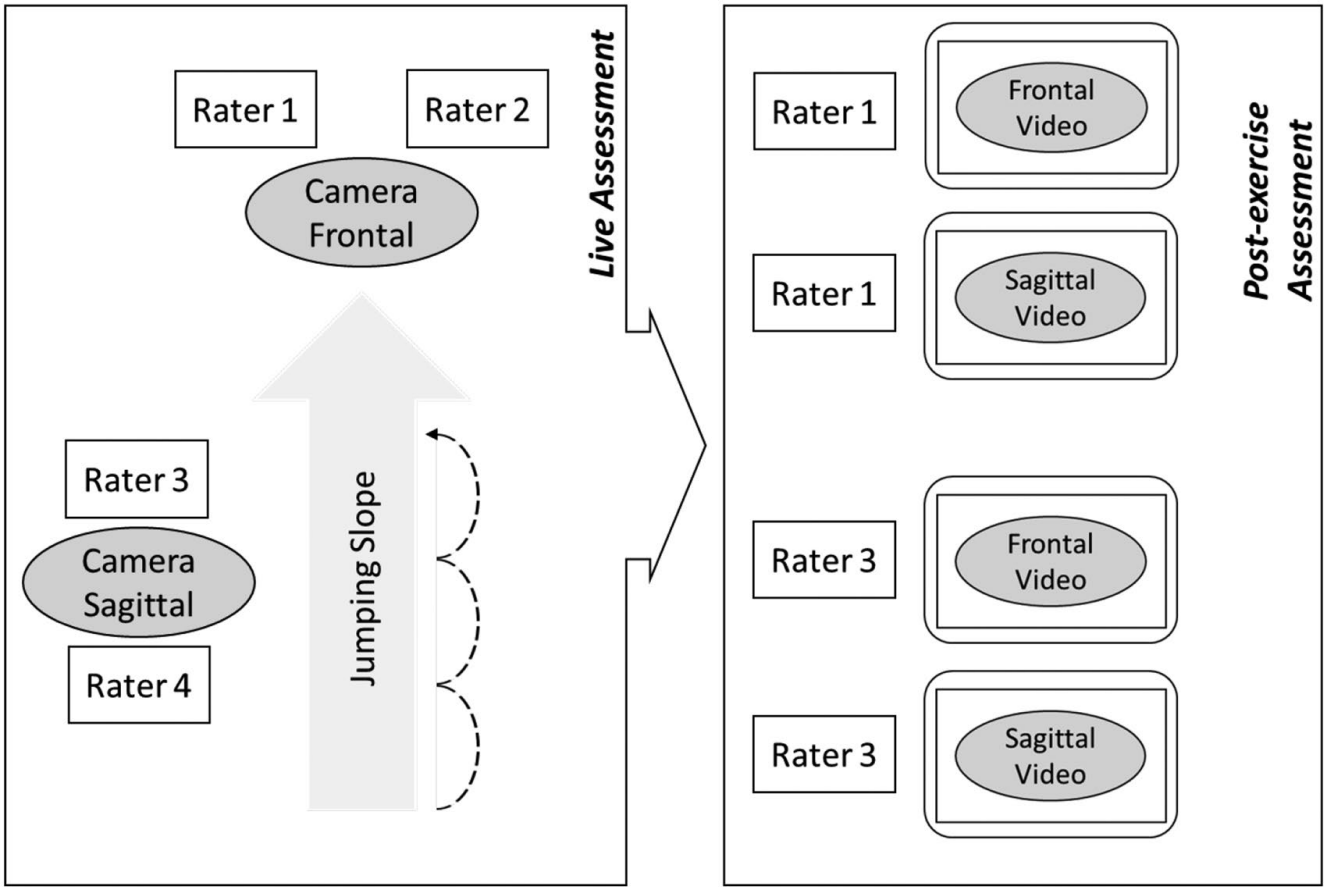
Measurement setup, with raters 1 and 2 as live raters from the frontal perspective, raters 3 and 4 as live raters from the sagittal perspective, and raters 1 and 3, who graded the quality of the movement execution post-exercise based on the video recordings from the frontal and sagittal perspectives, respectively.

The measurement setup is presented in Figure 1. For study aims 1 and 2, the post-exercise ratings of rater 1 (frontal perspective and based on the video recordings) were used for any further analysis.

#### Reliability of the live and post-exercise quality gradings

To evaluate the reliability of the proposed test approach, a total of four raters graded the quality of executing two-legged forward triple jumps. As part of the live grading, two raters assigned grades from a frontal perspective (rater 1 and rater 2), and two raters assigned grades from a sagittal perspective (rater 3 and rater 4). Video-based post-exercise grading was performed by two raters (rater 1 and rater 3, according to Figure 1). The criteria catalogue from the live assessment was adopted for post-exercise grading; however, no limitations were prescribed regarding the use of slow motion, still image extraction, or number of repetitions. All the participants were analysed by both raters from frontal and sagittal perspectives separately in a randomized order. Thus, a total of eight ratings were obtained from the analysis: four live ratings from four different raters, two per perspective, four post-exercise ratings from two raters (rater 1 and rater 3), each rater from both the frontal and sagittal perspectives.

### Statistical analysis

All the statistical analyses were performed using IBM SPSS statistics software version 28 and R Studio. For the variables age, height, weight, maturity offset and jump distance (both overall and regarding age, sex and grade subgroups), the assumption of normality was checked using the Shapiro–Wilk test and graphical parameters. Baseline characteristics and jump distance data are presented as the mean values and 95% confidence intervals. Differences between the groups/subgroups were identified by non-overlapping 95% confidence intervals and were backed up by unpaired sample t tests. Proportion differences in movement quality grades are presented as pie charts and were tested by Pearson χ^2^ tests. To analyse the differences in jump distance between the fixed factors sex (female vs. male) and grade (1, 2, 3), multivariate analyses of variance (MANOVAs) were used, with two separate analyses for the U16 and U11 age groups. Pairwise comparisons were performed using Bonferroni correction. Inter-rater grading reliability and live-to post-exercise grading agreement were assessed with Kendall’s coefficient of concordance (Kendall’s W). The following categorisation was adopted from Schmidt: W 0.1, *very weak agreement*; W 0.3, *weak agreement*; W 0.5, *moderate agreement*; W 0.7, *strong agreement*; and W 0.9, *unusually strong agreement* [27] to derive confidence in ranks. Inter-rater grading reliability was calculated for the sagittal and frontal ratings separately for both live and post-exercise gradings. Live-to post-exercise grading agreement was based on data from the frontal (rater 1) and sagittal (rater 3) perspectives, comparing their individual live and post-exercise gradings. The significance was set at α = 0.05.

## Results

### Baseline characteristics, jump performance and movement quality patterns according to age and sex

An overview of the participants’ age, height, weight, and maturity offset is presented in Table 1. All the measures showed differences between the U11 and U16 age groups in both sexes. Sex differences were only found for height in the U16 age group. Male U16 skiers were 5.4 cm taller than female U16 skiers.

**Table 1.** Baseline characteristics are presented as the means and 95% CIs in brackets.

	*Female*		*Male*	
	*U11*	*U16*	*U11*	*U16*
*n*	76	85	76	64
*Age (y)*	9.65* (9.44 − 9.86)	13.51* (13.24 − 13.79)	9.41* (9.20 − 9.62)	13.65* (13.36 − 13.95)
*Height (cm)*	137.2* (135.1 − 139.2)	156.9*# (155.1 − 158.6)	137.2* (135.4 − 139.0)	162.3*# (159.2 − 165.3)
*Weight (kg)*	31.6* (30.5 − 33.2)	48.0* (45.9 − 50.1)	30.4* (29.3 − 31.4)	50.9* (47.9 − 53.9)
*Maturity Offset (y)*	−3.79* (–3.95 – –3.63)	−0.87* (–1.12 – –0.62)	−3.92* (–4.07 – –3.77)	−0.63* (–0.96 – –0.30)

Differences were defined as non-overlapping cis and were backed-up by unpaired sample t tests; significant differences, which are depicted with ^*^for inter-age differences within the same sex and ^#^for inter-sex differences within the same age groups.

Jump distances for the U11 and U16 age groups of male and female skiers are presented in Table 2. In both sexes, U16 skiers had greater jump distances than U11 skiers did (0.98 and 1.46 m, for female and male skiers, respectively). Additionally, within the U16 age group, males jumped 0.53 m farther than female skiers did; however, there were no sex differences at the U11 level.

**Table 2.** Jump distance presented as the mean and 95% CI in brackets.

	*Female*		*Male*	
	*U11*	*U16*	*U11*	*U16*
*n*	76	85	76	64
*Jump Distance (m)*	4.39* (4.26 − 4.53)	5.37*# (5.21 − 5.53)	4.44* (4.33 − 4.55)	5.90*# (5.69 − 6.10)

Differences were defined as nonoverlapping cis and were backed-up by unpaired sample t tests, which are depicted with * for inter-age differences within the same sex and # for inter-sex differences within the same age groups.

**Figure 2.**
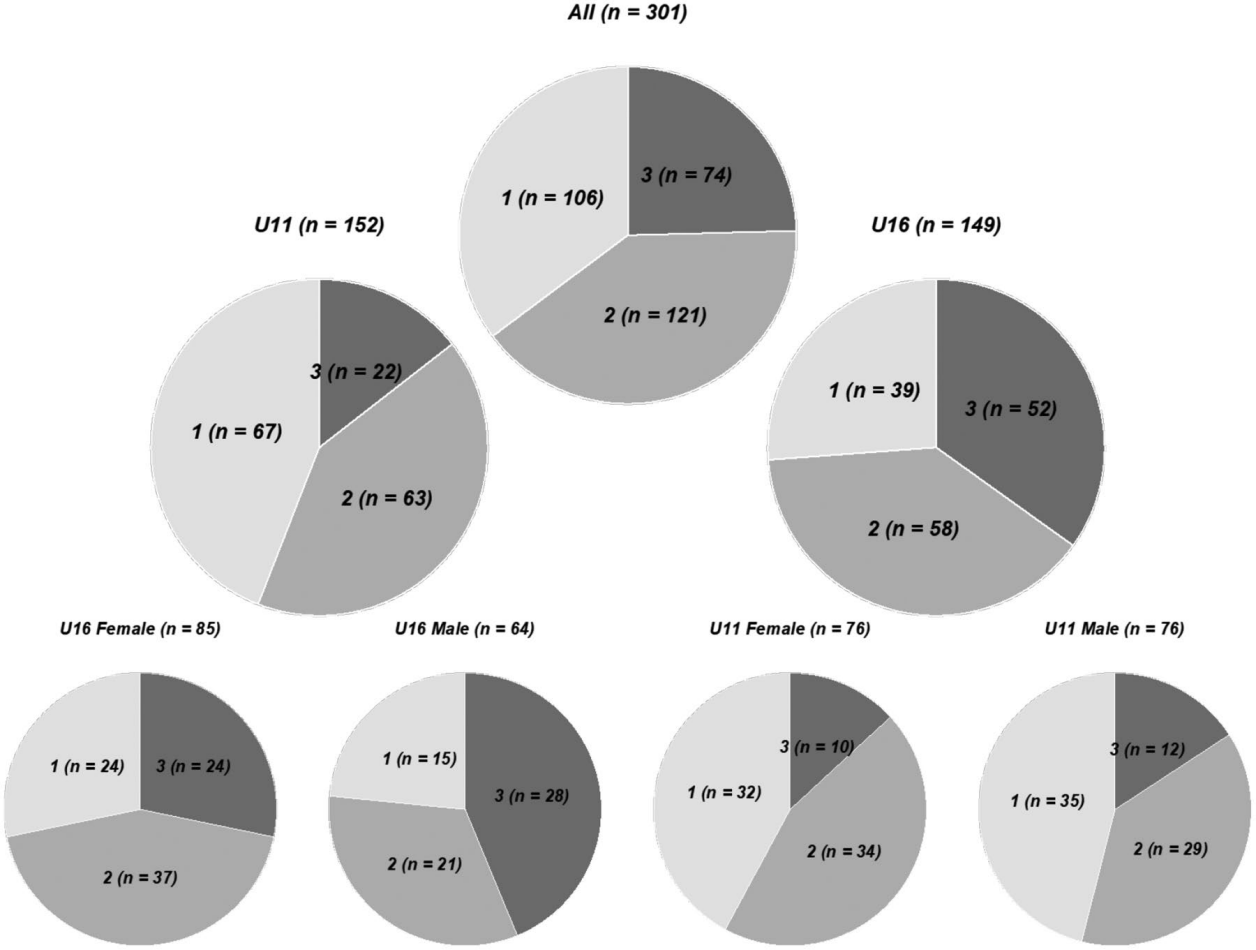
Pie charts presenting the distribution of the grades for all skiers and in the U11 and U16 groups stratified by sex. Grey scales from light to medium to dark representing the grades from 1 to 2 to 3.

The distributions of the grades for all skiers and in the U11 and U16 groups stratified by sex are shown in Figure 2. Pearson χ^2^ tests revealed significant differences in the distribution of movement quality grades between the U11 and U16 age groups (χ^2^(2) = 6.552, *p* = 0.038). Among both female and male skiers, there were more *good* execution quality (grade 3) and less *poor* execution quality (grade 1) ratings in the U16 group than in the U11 group (χ^2^(2) = 0.713, *p* = 0.700 and χ^2^(2) = 14.760, *p* < 0.001, respectively). No significant differences were found between the sexes in the U11 or U16 age groups (χ^2^(2) = 0.713, *p* = 0.700, and χ^2^(2) = 3.916, *p* = 0.141, respectively).

Table 3A+B shows the jump distances for the U11 and U16 groups stratified by sex and movement quality grades (1, 2, 3).

**Table 3.** A + B: Jump Distances for the U11 and U16 skiers stratified by sex and movement quality grade.

*U11 (n = 152)*							A
	*Female (n = 76)*			*Male (n = 76)*			
*Grade*	1 (*n* = 32)	2 (*n* = 34)	3 (*n* = 10)	1 (*n* = 35)	2 (*n* = 29)	3 (*n* = 12)	
*Jump Distance (m)*	4.30 (4.07 − 4.53)	4.46 (4.26 − 4.66)	4.46 (4.15 − 4.77)	4.46 (4.29 − 4.63)	4.37 (4.20 − 4.54)	4.55 (4.25 − 4.86)	
*U16 (n = 149)*							B
	*Female (n = 85)*			*Male (n = 64)*			
*Grade*	1 (*n* = 24)	2 (*n* = 37)	3 (*n* = 24)	1 (*n* = 15)	2 (*n* = 21)	3 (*n* = 28)	
*Jump Distance (m)*	4.94 (4.61 − 5.27)	5.42 (5.20 − 5.65)	5.72 (5.51–5.93)	5.67 (5.25 − 6.09)	5.61 (5.28 − 5.95)	6.23 (5.91 − 6.55)	

The data are presented as the means with 95% CIs in brackets.

On a multivariate level, a MANOVA for U11 skiers revealed no significant differences in jump distance between the sexes and grades at *p* < 0.05. There was also no interaction effect of sex*grading. For U16 skiers, another MANOVA revealed significant differences in jump distance between female and male subjects (*F* = 15.230, *p* < 0.001) and between skiers with different quality grades (*F* = 10.379, *p* < 0.001); however, there was no interaction effect between the factors sex and quality grade.

Post hoc pairwise comparisons in U16 skiers revealed a 0.77 m shorter jump distance in skiers with *poor* execution quality (i.e. grade 1) than in skiers with *good* execution quality (grade 3) (*p* < 0.001). Moreover, the jump distance was 0.50 m shorter for skiers with *reduced* execution quality (i.e. grade 2), than for skiers with *good* execution quality (i.e. grade 3) (*p* = 0.001).

### Interrater grading reliability and live-to post-exercise grading agreement

Table 4 shows the results from the agreement analysis. Inter-rater reliability for execution quality grading was found to be *moderate* for live ratings from both frontal and sagittal perspectives. Post-exercise grading showed *strong* inter-rater reliability for the frontal perspective and *moderate* inter-rater reliability for the sagittal perspectives. The live- to post-exercise grading agreement was *moderate* for the frontal perspective and *weak* for the *sagittal* perspective.

**Table 4.** Inter-rater grading reliability and live- to post-exercise grading agreement based on Kendall’s coefficient of concordance (Kendall’s W).

			Kendall’s W	Agreement	*p* Value
Inter-Rater Grading Reliability	Live	Frontal	0.566	moderate	0.058
		Sagittal	0.575	moderate	0.037
	Post	Frontal	0.726	strong	<0.001
		Sagittal	0.693	moderate	<0.001
Live to Post-Exercise Grading Agreement	Live - Post	Frontal	0.62	moderate	0.003
		Sagittal	0.489	weak	0.598

## Discussion

The main findings of this study were as follows: (1) jump distance was significantly greater in U16 skiers than in U11 skiers of both sexes and in skiers with good execution quality than in those with reduced or poor execution quality; (2) there were significant differences in jump distance between male and female skiers; these differences occurred mainly in the U16 skiers but not in the U11 skiers; and (3) slightly better inter-rater grading reliability was observed for video-based post-exercise ratings than for live ratings.

### Jump performance and movement quality patterns depending on age and sex

Jump distance was greater in the U16 age group than in the U11 age group. Interestingly, while in the U11 age group, the jump distance did not differ between male and female skiers, in the U16 age group, male skiers reached greater jump distances than females did. This aligns with the fact that hormone-related sex-specific differences in physical performance start at the age of 12–13 years [28]. Moreover, this highlights the sex-related developmental differences that occur around the age of 12 years, and therefore, a recommendation for differentiated training regimes in pre-pubertal skiers derives.

Similarly, the proportions of movement quality patterns differed between the U11 and U16 age groups. In particular, older skiers more often received the highest grade 3, representing good execution quality. This may be explained by an increase in motor control and strength during maturation around age at peak height velocity, which is commonly observed in pre-pubertal athletes [29]. Again, no difference in movement execution quality grades was detected in the U11 age group, and the distribution of movement execution quality grades did not differ between the sexes.

### Jump distance differs among skiers with different movement quality grades

Analysing the different groups that are formed according to movement execution grade, an increase in jump distance was observed with an increase in movement quality. There was a significant difference in jump distance between skiers in the U16 age group with the poorest grade (grade 1) and those in the best grade (grade 3). This could, to some degree, be explained through favourable movement patterns, with more efficient force transmissions. However, potential rater bias should be considered, and more powerful movement execution can lead to an impression of superior performance in terms of the leg axis.

### Inter-rater grading reliability and agreement between live and video-based post-exercise grading

Post-exercise quality grading based on video sequences of the test showed *moderate* and *strong* agreement from sagittal and frontal perspectives and thus slightly better inter-rater reliability than live ratings. A comparable study, however, including fewer dynamic exercises, revealed fair to high intra-rater reliability for a video-based movement quality assessment [30]. Accordingly, if feasible, a video-based post-exercise evaluation is recommended because it allows for greater confidence in the grading.

In principle, achieving *moderate* agreement is a rather problematic point for the practical implementation of this test. However, since it represents a feasible and meaningful holistic testing approach that can be used for the detection of individuals with deficits in motor control, for larger youth sport settings, it may nevertheless be justifiable. In particular, the three repetitions in a competitive setting are a large strength of the test. On the one hand, the complexity of the execution is high through threefold repetition, thus revealing potential deficits.

In addition, due to the competitive nature of the test, the test subjects were focused on maximum jump distances, resulting in maximal power during execution, again emphasising potential deficits. On the other hand, three repetitions allow the raters more data to be assigned a grade.

Overall, the two-legged forward triple jump test represents and may be considered a valuable and sufficiently reliable test approach for the detection of potential movement control deficiencies in large youth athlete cohorts. Moreover, through its simplicity in application, it could also be performed on a regular basis in training sessions and could, for example, deliver valuable information on long-term development during pre-pubertal stages. However, when high accuracy and precision are required or when cohorts are smaller and resources available, the use of a three-dimensional motion capture system rather than a simple visual quality assessment should be the first choice.

### Limitations and future research

As with all studies, our investigation has several limitations that should be mentioned. One of the weaknesses of cross-sectional studies is the inability to make causal inferences. Furthermore, in these types of studies, a sample of subjects from a large and heterogeneous population often needs to be selected, increasing the susceptibility of the results to sampling bias [31]. Awareness of these limitations requires, for example, specific considerations of the analytical approach. Our study aimed to establish the reliability and feasibility of a forward-jumping movement quality rating; however, having only three grades might not be sufficient for a comprehensive rating. Thus, the extension of the grading scale to five grades can be considered to maintain high feasibility but increase sensitivity. Moreover, the incorporation of frontal and sagittal perspective grading is a strength of this test; however, a suitable approach to merge both into one representative grade should be developed.

## Conclusion

In young competitive alpine skiers, jump performance increases with age, and sex differences begin to manifest upon reaching puberty. Moreover, our results emphasise the necessity of assessing both jumping distance and movement quality in this age group, not only for injury prevention reasons, which are well described in the literature but also, as shown here, for improving jumping performance. In this regard, a test battery that focuses on both jump distance and movement quality might help to identify athletes with specific training needs. Post-exercise video-based quality grading should be considered to improve inter-rater reliability where resources are available.

## Data Availability

Restrictions apply to the availability of these data. Data were obtained from Swiss-Ski and are available from the authors with the permission of Swiss-Ski.
